# Supplementary material for: Mycobacterium tuberculosis suppresses host antimicrobial peptides by dehydrogenating L-alanine
Source: Nat Commun. 2024 May 17;15:4216. doi: 10.1038/s41467-024-48588-4 (PMC11101664; doi:10.1038/s41467-024-48588-4)

Supplementary Information

Mycobacterium tuberculosis suppresses host antimicrobial peptides by dehydrogenating L-alanine

Cheng Peng[1,2,5], Yuanna Cheng[1,2,5], Mingtong Ma[1,2,5], Qiu Chen[1,2], Yongjia Duan[1,2], Shanshan Liu[1,2], Hongyu Cheng[1,2], Hua Yang[1,3], Jingping Huang[1,2], Wenyi Bu[1,2], Chenyue Shi[1,2], Xiangyang Wu[1,4], Jianxia Chen[1,3,4], Ruijuan Zheng[1,3], Zhonghua Liu[1,3], Zhe Ji[2], Jie Wang[1,3], Xiaochen Huang[1,3], Peng Wang[3], Wei Sha[3], Baoxue Ge[1,2,3,4*] and Lin Wang[1,2,3*]

[1]Shanghai Key Laboratory of Tuberculosis, Shanghai Pulmonary Hospital, Tongji University School of Medicine; Shanghai, China.

[2]Department of Microbiology and Immunology, Tongji University School of Medicine; Shanghai, China.

[3]Shanghai Clinic and Research Center of Tuberculosis, Shanghai Pulmonary Hospital, Tongji University School of Medicine; Shanghai, China.

[4]Clinical Translation Research Center, Shanghai Pulmonary Hospital, Tongji University School of Medicine; Shanghai, China.

[5]These authors contributed equally to this work.

*Correspondence should be addressed to: Lin Wang (651377481@qq.com); Baoxue Ge (gebaoxue@sibs.ac.cn)

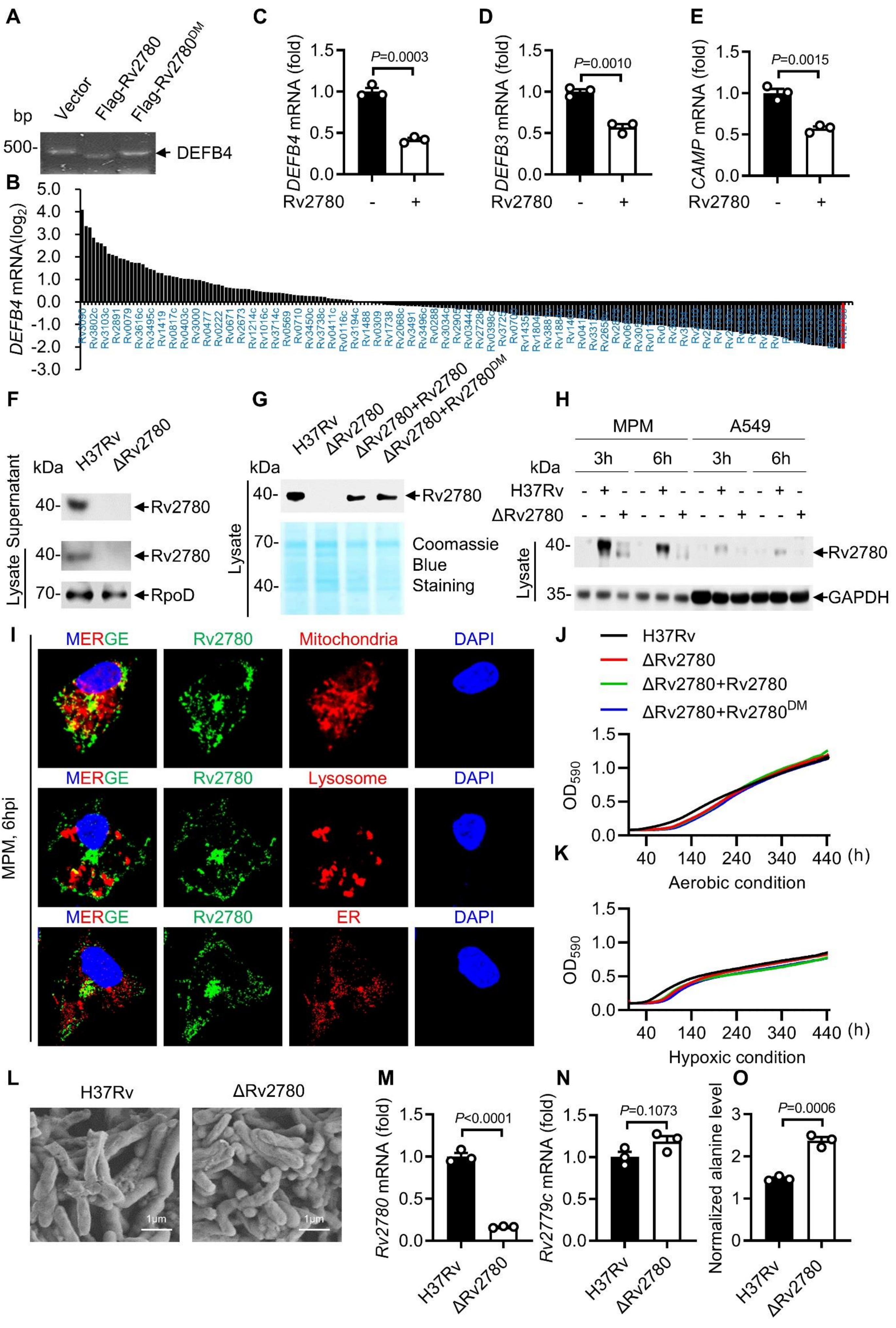
A
bp
Vector
Flag-Rv2780
Flag-Rv2780DM
500-
DEFB4
B
DEFB4 mRNA(log2)
C
DEFB4 mRNA (fold)
P=0.0003
Rv2780
D
DEFB3 mRNA (fold)
P=0.0010
E
CAMP mRNA (fold)
P=0.0015
F
kDa
H37Rv
ΔRv2780
Supernatant
Lysate
Rv2780
RpoD
G
ΔRv2780+Rv2780
ΔRv2780+Rv2780DM
Coomassie
Blue
Staining
H
MPM
A549
3h
6h
GAPDH
I
MERGE
Mitochondria
DAPI
Lysosome
ER
MPM, 6hpi
J
OD590
Aerobic condition
K
Hypoxic condition
L
1μm
M
Rv2780 mRNA (fold)
P<0.0001
N
Rv2779c mRNA (fold)
P=0.1073
O
Normalized alanine level
P=0.0006

Supplementary Fig. 1 Rv2780 inhibits antimicrobial peptides. (**A**) Agarose gel electrophoresis for *DEFB4* gene amplified from cDNA clones of HEK293T cells transfected with empty vector, plasmids encoding *M. tuberculosis* Rv2780 or dehydrogenase-inactive mutant of Rv2780 ($Rv2780^{DM}$). (**B**) RT-PCR analysis of *DEFB4* in HEK293T cells transfected with empty vector or plasmids encoding 201 *M. tuberculosis* secreted proteins or lipoproteins independently. The red column represents Rv2780. (**C-E**) RT-PCR analysis of *DEFB4* (**C**), *DEFB3* (**D**) and *CAMP* (**E**) in HEK293T cells transfected with empty vector (Black) or vector encoding *M. tuberculosis* Rv2780 (White) for 24 hours. (**F-G**) Immunoblot of supernatant or lysates from H37Rv, H37RvΔRv2780, H37Rv (ΔRv2780+Rv2780) and H37Rv (ΔRv2780+$Rv2780^{DM}$) strains using anti-Rv2780 antibody. Anti-RpoD antibody was used as control. (**H**) Immunoblot analysis of Rv2780 in cell lysates of mice peritoneal macrophages or A549 cells infected with H37Rv or H37RvΔRv2780 for 3 and 6 hours (MOI=2). (**I**) Immunofluorescence analysis of Rv2780 and subcellular staining (mitochondria, lysosome or ER) in mice peritoneal macrophages at 6 hours post infection (hpi) (MOI=2). (**J-K**) Growth curve of H37Rv, H37RvΔRv2780, H37Rv (ΔRv2780+Rv2780) and H37Rv (ΔRv2780+$Rv2780^{DM}$) strains cultured in aerobic condition (**J**) and hypoxic condition (**K**). (**L**) Scanning electron microscope images of wild-type H37Rv and H37RvΔRv2780 strains. (**M-N**) RT-PCR analysis of *Rv2780* (**M**) and *Rv2779c* (**N**) in wild-type H37Rv and H37RvΔRv2780 strains. (**O**) Alanine detection assay of H37Rv or H37RvΔRv2780 strains. Alanine level was normalized to protein level. Data in **B** are representative of one experiment, mean. Data except for **B** are representative of one experiment with at least three independent biological replicates; (**C-E** and **M-O**) $n = 3$, each circle represents one technical repeat (mean ± s.e.m); (**J** and **K**) (mean). Two-tailed unpaired Student's *t*-test (**C-E** and **M-O**) was used for statistical analysis. *P* values are shown in **C-E** and **M-O**. Source data are provided as a Source Data file.

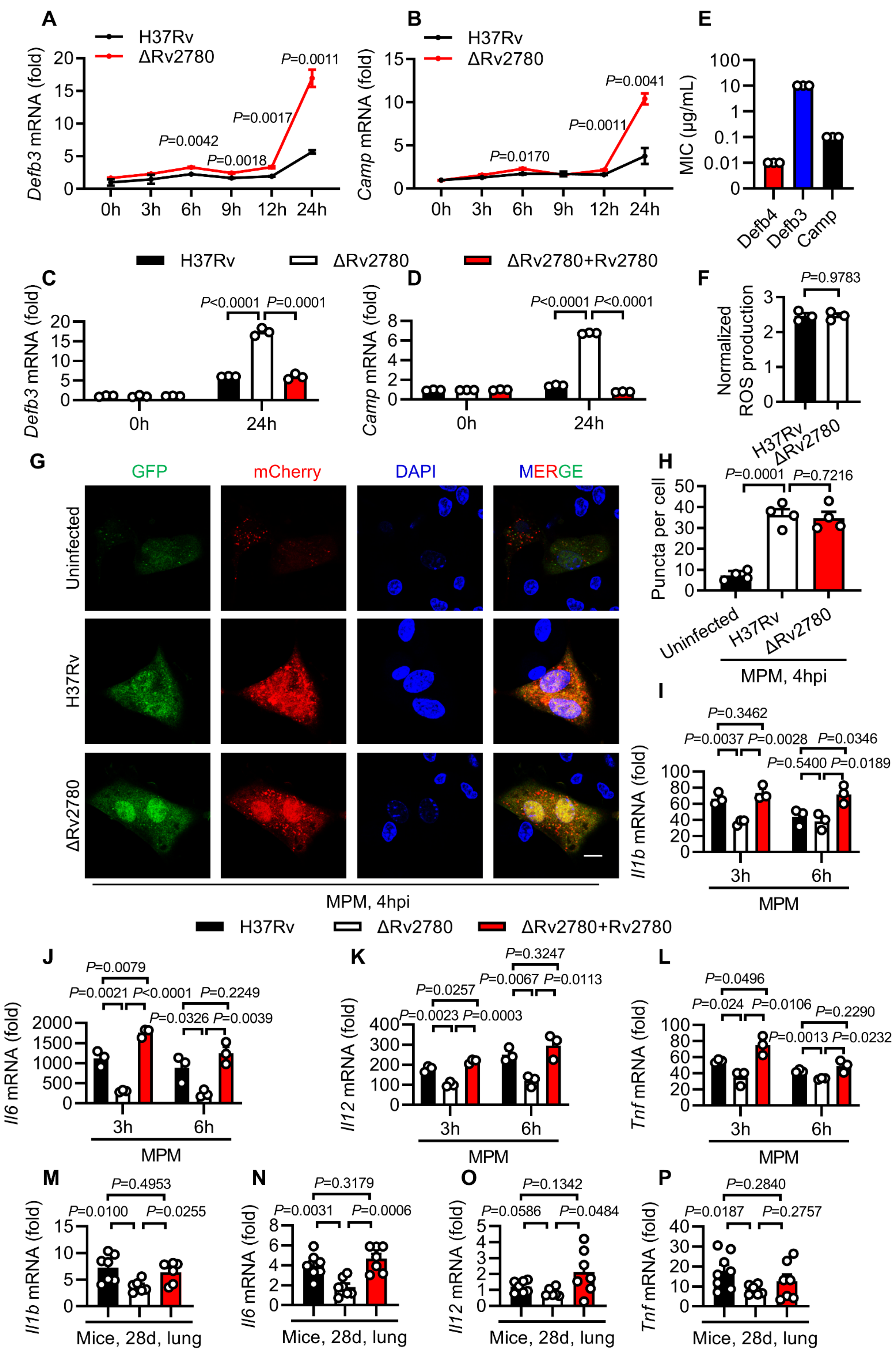

A
H37Rv
ΔRv2780
Defb3 mRNA (fold)
P=0.0042
P=0.0018
P=0.0017
P=0.0011
0h 3h 6h 9h 12h 24h
B
Camp mRNA (fold)
P=0.0170
P=0.0011
P=0.0041
E
MIC (μg/mL)
Defb4
Defb3
Camp
H37Rv
ΔRv2780
ΔRv2780+Rv2780
C
P<0.0001
P=0.0001
D
P<0.0001
P<0.0001
F
Normalized ROS production
P=0.9783
G
GFP
mCherry
DAPI
MERGE
Uninfected
H37Rv
ΔRv2780
MPM, 4hpi
H
Puncta per cell
P=0.0001
P=0.7216
Uninfected
MPM, 4hpi
I
Il1b mRNA (fold)
P=0.3462
P=0.0037
P=0.0028
P=0.0346
P=0.5400
P=0.0189
3h
6h
MPM
J
Il6 mRNA (fold)
P=0.0079
P=0.0021
P<0.0001
P=0.2249
P=0.0326
P=0.0039
K
Il12 mRNA (fold)
P=0.3247
P=0.0067
P=0.0113
P=0.0257
P=0.0023
P=0.0003
L
Tnf mRNA (fold)
P=0.0496
P=0.024
P=0.0106
P=0.2290
P=0.0013
P=0.0232
M
P=0.4953
P=0.0100
P=0.0255
Mice, 28d, lung
N
P=0.3179
P=0.0031
P=0.0006
O
P=0.1342
P=0.0586
P=0.0484
P
P=0.2840
P=0.0187
P=0.2757

Supplementary Fig. 2 Rv2780 dehydrogenates L-alanine. (**A-B**) RT-PCR analysis of *Defb3* (**A**) and *Camp* (**B**) in mice peritoneal macrophages infected with wild-type H37Rv or H37RvΔRv2780 for 0, 3, 6, 9,12 and 24 hours (MOI=2). (**C-D**) RT-PCR analysis of *Defb3* (**C**) and *Camp* (**D**) in mice peritoneal macrophages infected with wild-type H37Rv, H37RvΔRv2780 or H37Rv(ΔRv2780+Rv2780) for 0 and 24 hours (MOI=2). (**E**) Minimum Inhibitory Concentrations (MICs) of Camp, Defb4 and Defb3 against *Mycobacterium tuberculosis* H37Rv. (**F**) ROS quantification assay in mice peritoneal macrophages infected with H37Rv and H37RvΔRv2780 for 4 hours (MOI=2). (**G-H**) Imaging (**G**) and quantification(**H**) of the autophagic flux in mice peritoneal macrophages infected with adenovirus expressing mCherry-GFP-LC3B fusion protein at 4 hours post infection of H37Rv, H37RvΔRv2780 or H37Rv(ΔRv2780+Rv2780) (MOI=2). (**I-L**) RT-PCR analysis of *Il1b* (**I**), *Il6* (**J**), *Il12* (**K**) and *Tnf* (**L**) in mice peritoneal macrophages infected with wild-type H37Rv, H37RvΔRv2780 or H37Rv(ΔRv2780+Rv2780) for 3 and 6 hours (MOI=2). Data represent fold change of cytokines compared with uninfected group. (**M-P**) RT-PCR analysis of *Il1b* (**M**), *Il6* (**N**), *Il12* (**O**) and *Tnf* (**P**) in lung tissues of mice infected with wild-type H37Rv, H37RvΔRv2780 or H37Rv(ΔRv2780+Rv2780) for 28 days (MOI=2). Data in **I-P** represent fold change of cytokines compared with uninfected group. Data in **A-P** are representative of one experiment with at least three independent biological replicates; (**A-F** and **H-L**) $n = 3$, each circle represents one technical repeat (mean ± s.e.m); (**M-P**) $n = 6$ or $n = 7$ mice (mean ± s.e.m). Two-tailed unpaired Student's *t*-test (**A-D, F** and **H-L**) and two-sided Mann–Whitney *U*-test (**M-P**) were used for statistical analysis. *P* values are shown in **A-D**, **F** and **H-P**. Source data are provided as a Source Data file.

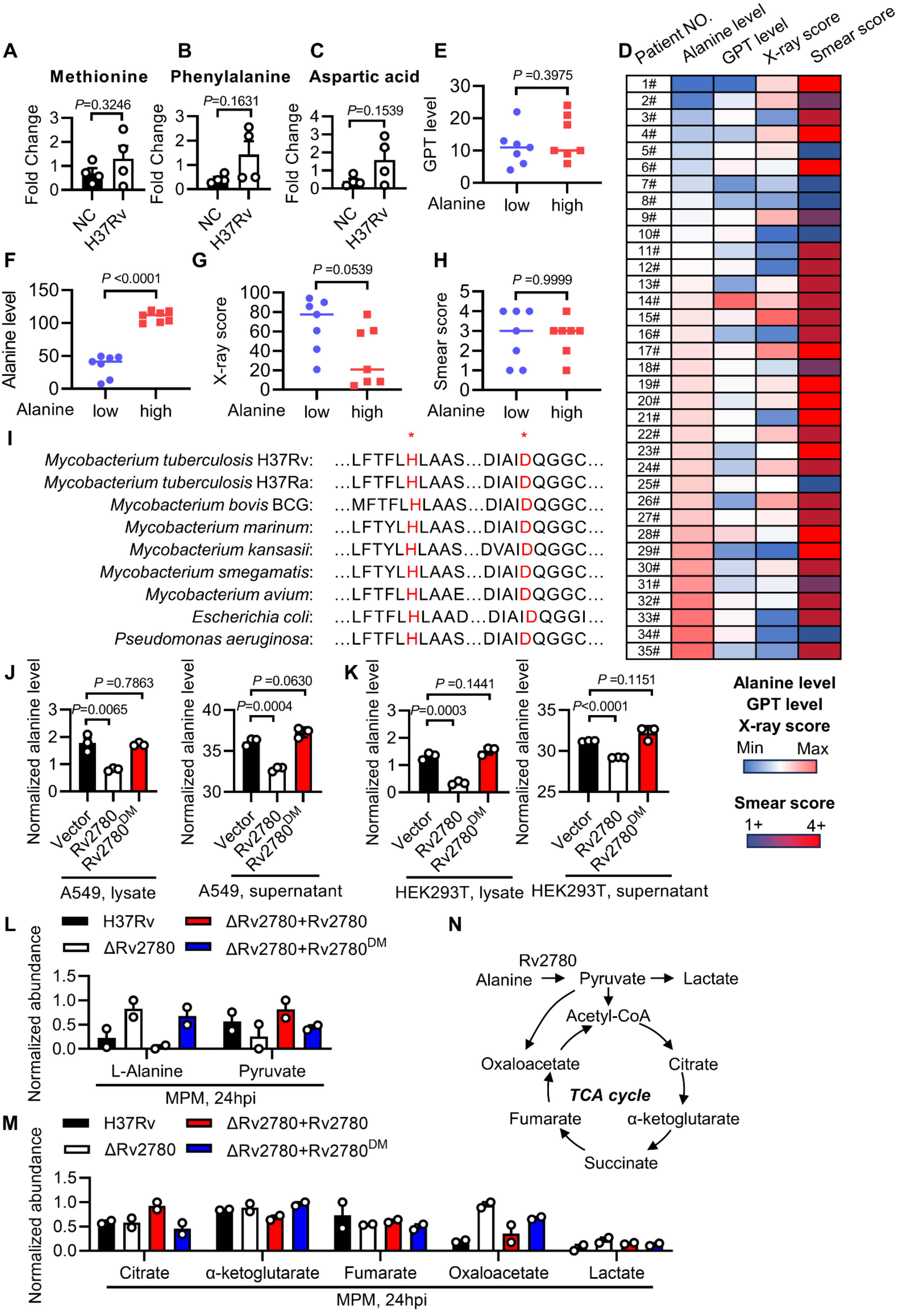
A
Methionine
P=0.3246
Fold Change
NC
H37Rv
B
Phenylalanine
P=0.1631
Fold Change
NC
H37Rv
C
Aspartic acid
P=0.1539
Fold Change
NC
H37Rv
E
P =0.3975
GPT level
Alanine low high
D
Patient NO.
Alanine level
GPT level
X-ray score
Smear score
1# 2# 3# 4# 5# 6# 7# 8# 9# 10# 11# 12# 13# 14# 15# 16# 17# 18# 19# 20# 21# 22# 23# 24# 25# 26# 27# 28# 29# 30# 31# 32# 33# 34# 35#
F
P <0.0001
Alanine level
Alanine low high
G
P =0.0539
X-ray score
Alanine low high
H
P =0.9999
Smear score
Alanine low high
I
Mycobacterium tuberculosis H37Rv: ...LFTFLHLAAS...DIAIDQGGC...
Mycobacterium tuberculosis H37Ra: ...LFTFLHLAAS...DIAIDQGGC...
Mycobacterium bovis BCG: ...MFTFLHLAAS...DIAIDQGGC...
Mycobacterium marinum: ...LFTYLHLAAS...DIAIDQGGC...
Mycobacterium kansasii: ...LFTYLHLAAS...DVAIDQGGC...
Mycobacterium smegamatis: ...LFTYLHLAAS...DIAIDQGGC...
Mycobacterium avium: ...LFTFLHLAAE...DIAIDQGGC...
Escherichia coli: ...LFTFLHLAAD...DIAIDQGGI...
Pseudomonas aeruginosa: ...LFTFLHLAAS...DIAIDQGGC...
J
Normalized alanine level
P =0.7863
P=0.0065
Vector Rv2780 Rv2780DM
A549, lysate
P =0.0630
P=0.0004
A549, supernatant
K
P =0.1441
P=0.0003
HEK293T, lysate
P =0.1151
P<0.0001
HEK293T, supernatant
Alanine level
GPT level
X-ray score
Min Max
Smear score
1+ 4+
L
H37Rv
ΔRv2780
ΔRv2780+Rv2780
ΔRv2780+Rv2780DM
Normalized abundance
L-Alanine
Pyruvate
MPM, 24hpi
N
Rv2780
Alanine
Pyruvate
Lactate
Acetyl-CoA
Oxaloacetate
Citrate
TCA cycle
Fumarate
α-ketoglutarate
Succinate
M
H37Rv
ΔRv2780
ΔRv2780+Rv2780
ΔRv2780+Rv2780DM
Normalized abundance
Citrate
α-ketoglutarate
Fumarate
Oxaloacetate
Lactate
MPM, 24hpi

Supplementary Fig. 3 Rv2780 suppress AMPs by dehydrogenating alanine. (**A-C**) GC-MS analysis of methionine (**A**), phenylalanine (**B**) and aspartic acid (**C**) in serum of uninfected mice (NC) and H37Rv-infected mice (H37Rv). (**D-H**) Heatmap (**D**) of normalized GPT level (**E**), alanine level (**F**), X-ray score (**G**) and smear score (**H**) in plasma from 35 TB patients. 1-35 represents 35 TB patients. (**I**) The conserved Rv2780 catalytic active sites in different bacterium. (**J-K**) Quantitative analysis of alanine in cell lysates and supernatants of A549 cells (**J**) or HEK293T cells (**K**) transfected with vector or plasmid encoding Rv2780 or enzymatic activity mutant Rv2780^{DM} for 24 hours. (**L-M**) Normalized abundance of alanine and pyruvate (**L**) and other pyruvate-associated metabolites (**M**) of in mice peritoneal macrophages infected with H37Rv, H37RvΔRv2780, H37Rv(ΔRv2780+Rv2780) or H37Rv(ΔRv2780+ Rv2780^{DM}) for 24 hours. (**N**) Representative metabolic pathway of alanine and pyruvate and tricarboxylic acid cycle (TCA cycle). Data in **J-K** are representative of one experiment with at least three independent biological replicates; (**A-C**) $n = 4$ mice (mean ± s.e.m); (**E-H**) $n = 7$ (mean ± s.e.m). Two-tailed unpaired Student's *t*-test (**J-K**) and two-sided Mann-Whitney *U*-test (**A-C** and **E-H**) were used for statistical analysis. *P* values are shown in **A-H** and **J-K**. Source data are provided as a Source Data file.

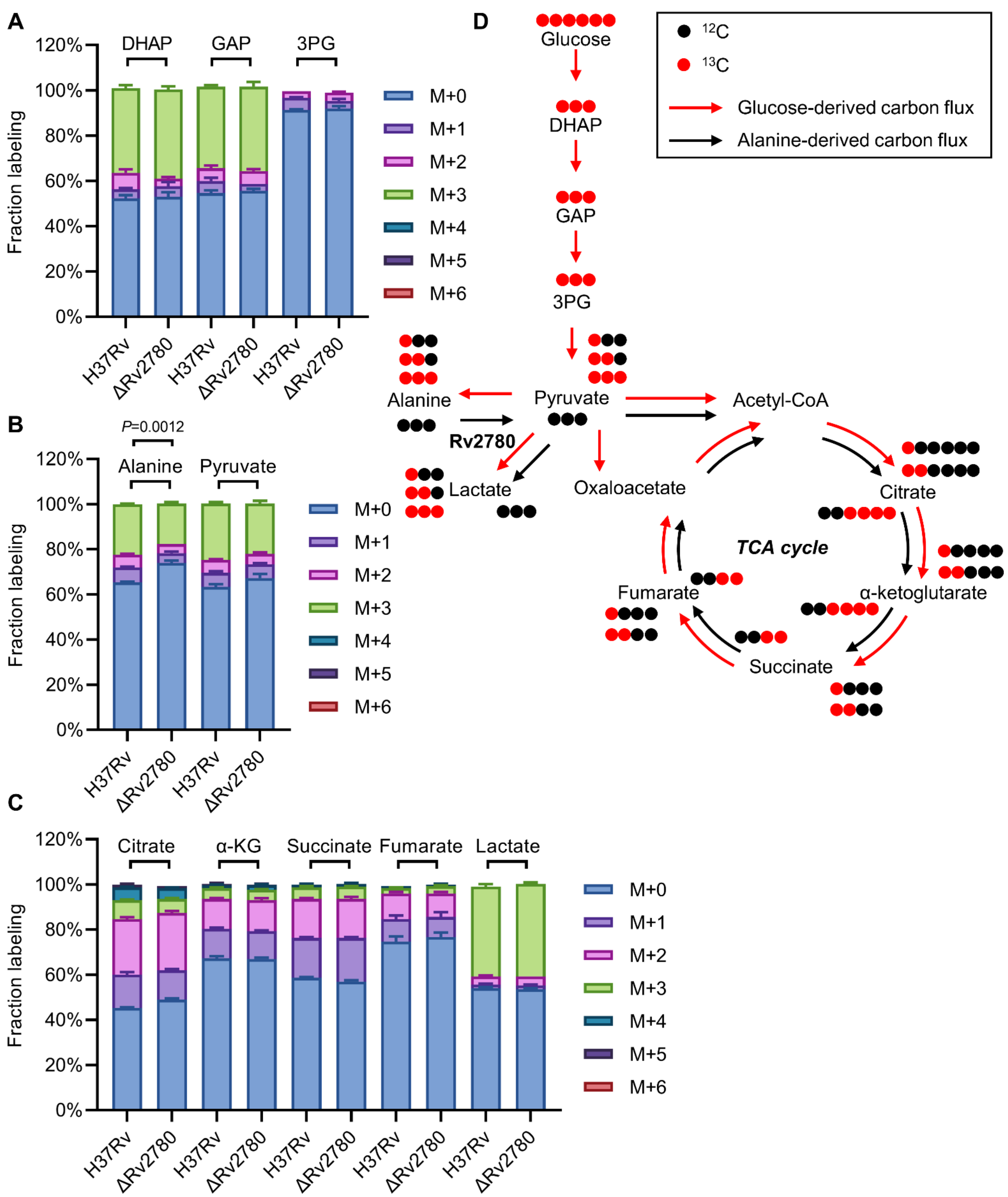

Supplementary Fig. 4 Carbon flux analysis. (**A-C**) Percentage of ^{13}C in different isotopologues of DHAP, GAP and 3PG (**A**); alanine and pyruvate (**B**); citrate, α-ketoglutarate (α-KG), succinate, fumarate and lactate (**C**). (**D**) Schematic illustration of atom transitions in central metabolism using uniformly labeled ^{13}C-glucose ([U-^{13}C]) (labeled carbons are indicated in red) as tracer for intracellular metabolic fluxes of macrophages infected with H37Rv or ΔRv2780 for 24 hours. DHAP, dihydroxyacetone phosphate; GAP, glyceraldehyde-

3-phosphate; 3PG, 3-phosphoglycerate. Two-tailed unpaired Student's *t*-test (**B**) was used for statistical analysis. *P* values are shown in **B**. Source data are provided as a Source Data file.

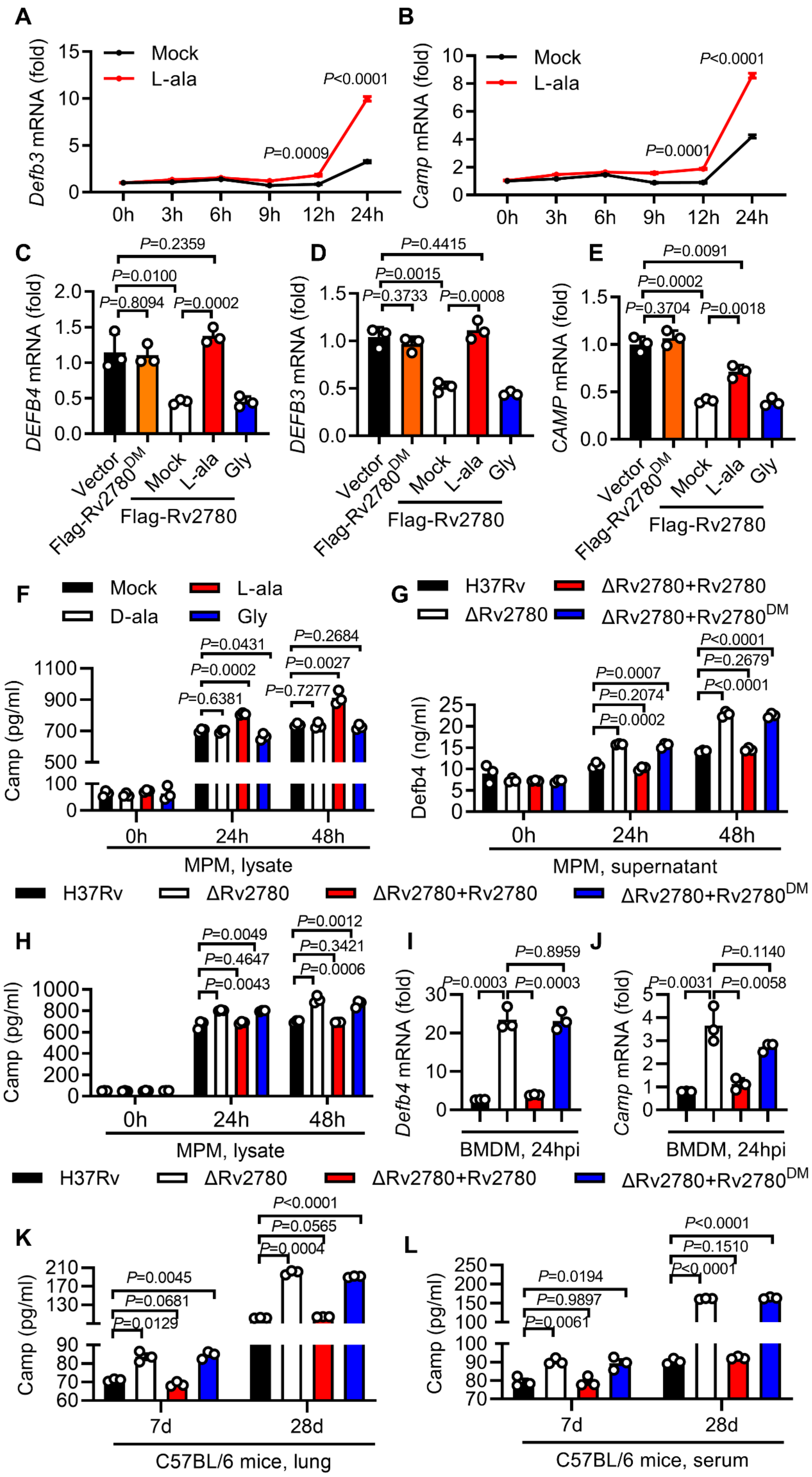
A
B
C
D
E
F
G
H
I
J
K
L
Mock
L-ala
Defb3 mRNA (fold)
Camp mRNA (fold)
P<0.0001
P=0.0009
P=0.0001
0h
3h
6h
9h
12h
24h
DEFB4 mRNA (fold)
DEFB3 mRNA (fold)
CAMP mRNA (fold)
Vector
Flag-Rv2780DM
Mock
L-ala
Gly
Flag-Rv2780
P=0.2359
P=0.0100
P=0.8094
P=0.0002
P=0.4415
P=0.0015
P=0.3733
P=0.0008
P=0.0091
P=0.0002
P=0.3704
P=0.0018
D-ala
Camp (pg/ml)
MPM, lysate
P=0.0431
P=0.0002
P=0.6381
P=0.2684
P=0.0027
P=0.7277
H37Rv
ΔRv2780
ΔRv2780+Rv2780
ΔRv2780+Rv2780DM
Defb4 (ng/ml)
MPM, supernatant
P=0.0007
P=0.2074
P=0.0002
P<0.0001
P=0.2679
P<0.0001
P=0.0049
P=0.4647
P=0.0043
P=0.0012
P=0.3421
P=0.0006
Defb4 mRNA (fold)
BMDM, 24hpi
P=0.8959
P=0.0003
P=0.0003
Camp mRNA (fold)
P=0.1140
P=0.0031
P=0.0058
C57BL/6 mice, lung
P=0.0045
P=0.0681
P=0.0129
P<0.0001
P=0.0565
P=0.0004
7d
28d
C57BL/6 mice, serum
P=0.0194
P=0.9897
P=0.0061
P<0.0001
P=0.1510
P<0.0001

Supplementary Fig. 5 L-alanine induce AMPs expression. (**A-B**) RT-PCR analysis of *Defb3* (**A**) and *Camp* (**B**) in mice peritoneal macrophages treated with 1 mM L-alanine for 12 hours followed by H37Rv infection for 0, 3, 6, 9, 12 and 24 hours (MOI=2). (**C-E**) RT-PCR analysis of *DEFB4* (**C**), *DEFB3* (**D**) and *CAMP* (**E**) in HEK293T cells treated with 1 mM L-alanine, 1 mM glycine, 1 mM L-isoleucine or transfected with Rv2780 or Rv2780^{DM} plasmids for 24 hours. (**F**) ELISA analysis of Camp in cell lysates of mice peritoneal macrophages treated with 1 mM D-alanine, 1 mM L-alanine or 1 mM glycine followed by H37Rv infection for 0, 24 and 48 hours (MOI=2). (**G-H**) ELISA analysis of Defb4 in supernatants (**G**) and Camp in cell lysates (**H**) of mice peritoneal macrophages infected with H37Rv, H37RvΔRv2780, H37Rv (ΔRv2780+Rv2780) and H37Rv (ΔRv2780+Rv2780^{DM}) strains for 0, 24 and 48 hours (MOI=2). (**I-J**) RT-PCR analysis of *Defb4* (**I**) and *Camp* (**J**) in BMDMs infected with H37Rv, H37RvΔRv2780, H37Rv (ΔRv2780+Rv2780) and H37Rv (ΔRv2780+Rv2780^{DM}) strains for 24 hours (MOI=2). (**K-L**) ELISA analysis of Camp in lung homogenates (**K**) and serum (**L**) or of mice infected with indicated strains for 7 and 28 days. Data in **I-L** represent fold change of AMPs compared with uninfected group. Data are representative of one experiment with at least three independent biological replicates; (**A-J**) $n = 3$, each circle in **C-J** represents one technical repeat (mean ± s.e.m); (**K-L**) $n = 3$ mice (mean ± s.e.m). Two-tailed unpaired Student's *t*-test (**A-J**) and two-sided Mann–Whitney *U*-test (**K-L**) were used for statistical analysis. *P* values are shown in **A-L**. Source data are provided as a Source Data file.

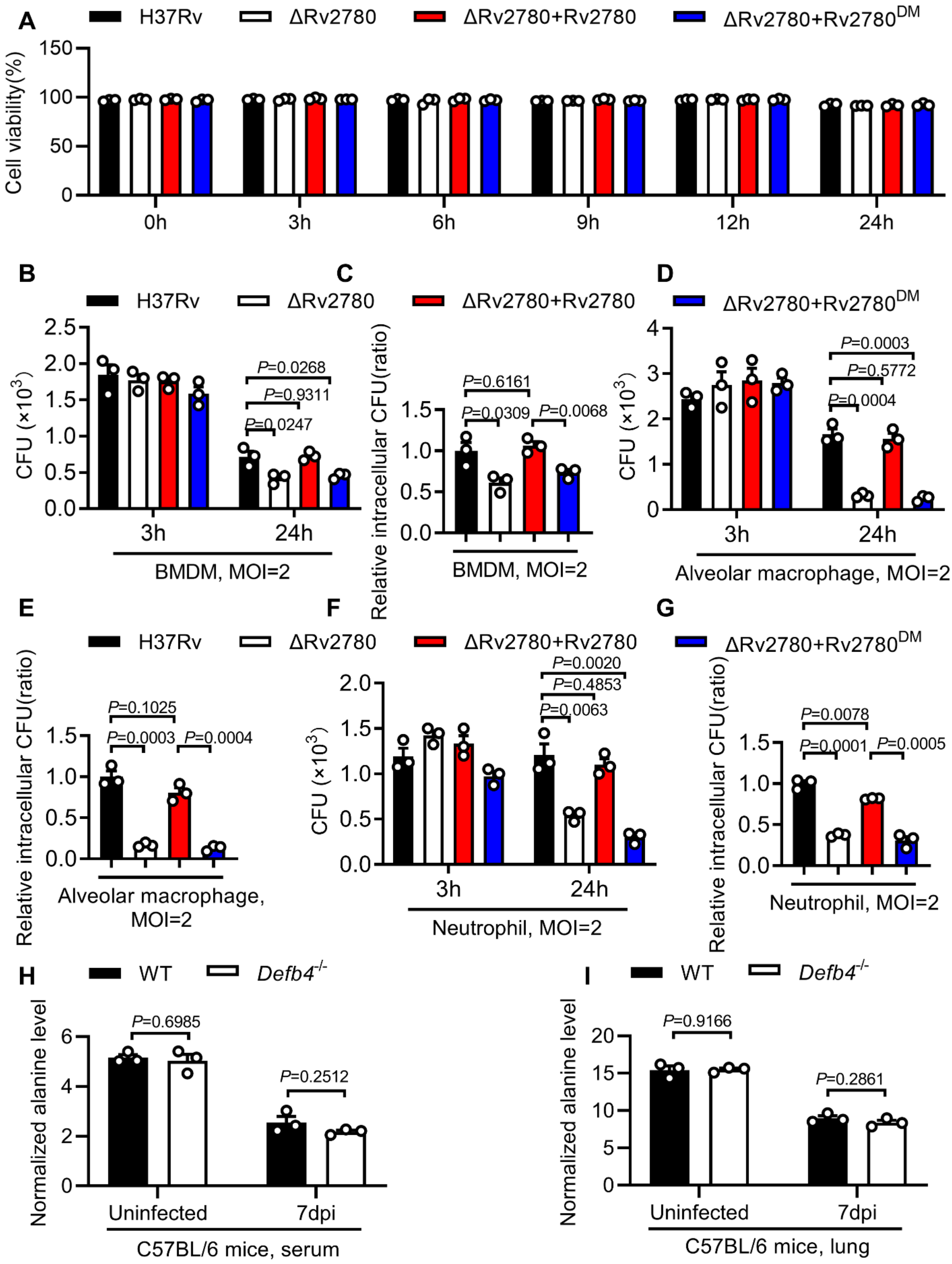

Supplementary Fig. 6 Rv2780 promotes intracellular *M. tuberculosis* survival via dehydrogenase activity. (**A**) MTT assay of cell viability from mice peritoneal macrophages infected with indicated strains for 0, 3, 6, 9, 12 and 24 hours. (**B-C**) CFU counts (**B**) and relative intracellular CFU ratio (**C**) in mice bone marrow-derived macrophages (BMDMs) infected with H37Rv, H37RvΔRv2780, H37Rv(ΔRv2780+Rv2780) or H37Rv(ΔRv2780+Rv2780^{DM}) strains for 3 and 24 hours (MOI=2). (**D-E**) CFU counts (**D**) and relative

intracellular CFU ratio (**E**) in mice alveolar macrophages infected with H37Rv, H37RvΔRv2780, H37Rv(ΔRv2780+Rv2780) or H37Rv(ΔRv2780+Rv2780^{DM}) strains for 3 and 24 hours (MOI=2). (**F-G**) CFU counts (**F**) and relative intracellular CFU ratio (**G**) in mice bone marrow derived neutrophils infected with H37Rv, H37RvΔRv2780, H37Rv(ΔRv2780+Rv2780) or H37Rv(ΔRv2780+Rv2780^{DM}) strains for 3 and 24 hours (MOI=2). (**H-I**) Quantitative analysis of alanine in serum (**H**) and lung homogenate (**I**) of wild type and Defb4$^{-/-}$ mice infected for 7 days. Data are representative of one experiment with at least three independent biological replicates; (**A-G**) $n = 3$, each circle represents one technical repeat (mean ± s.e.m); (**H-I**) $n = 3$ mice (mean ± s.e.m). Two-tailed unpaired Student's *t*-test (**A-G**) and two-sided Mann-Whitney *U*-test (**H-I**) were used for statistical analysis. *P* values are shown in **B-I**. Source data are provided as a Source Data file.

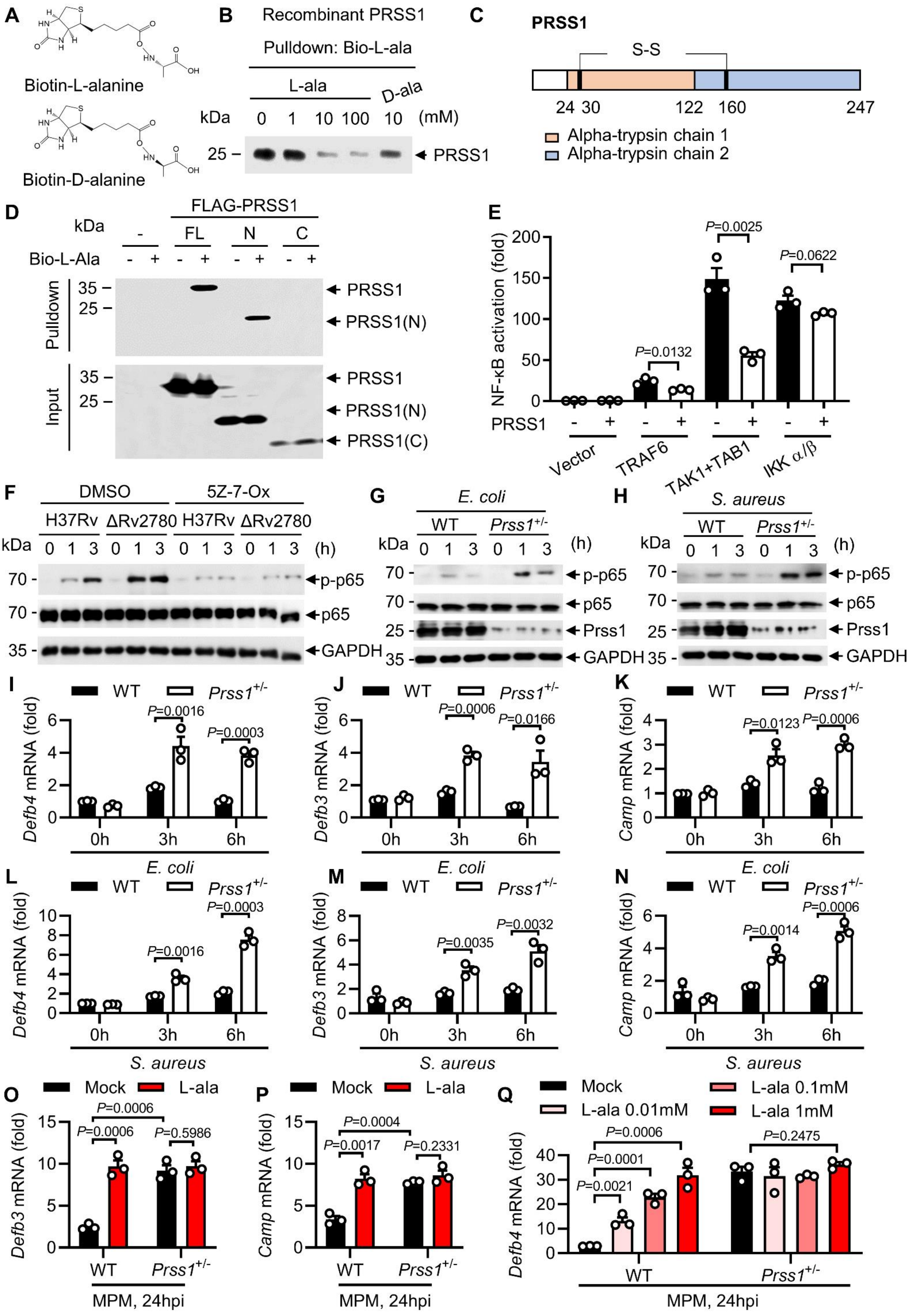

A
Biotin-L-alanine
Biotin-D-alanine
B
Recombinant PRSS1
Pulldown: Bio-L-ala
L-ala
D-ala
kDa
0 1 10 100 10 (mM)
25 –
PRSS1
C
PRSS1
S-S
24 30 122 160 247
Alpha-trypsin chain 1
Alpha-trypsin chain 2
D
FLAG-PRSS1
kDa
- FL N C
Bio-L-Ala
Pulldown
Input
35 –
25 –
PRSS1
PRSS1(N)
PRSS1(C)
E
NF-κB activation (fold)
P=0.0025
P=0.0622
P=0.0132
PRSS1
Vector
TRAF6
TAK1+TAB1
IKK α/β
F
DMSO
5Z-7-Ox
H37Rv
ΔRv2780
kDa
0 1 3 (h)
p-p65
p65
GAPDH
G
E. coli
WT
Prss1+/-
p-p65
p65
Prss1
GAPDH
H
S. aureus
WT
Prss1+/-
p-p65
p65
Prss1
GAPDH
I
J
K
WT
Prss1+/-
Defb4 mRNA (fold)
Defb3 mRNA (fold)
Camp mRNA (fold)
P=0.0016
P=0.0003
P=0.0006
P=0.0166
P=0.0123
P=0.0006
0h 3h 6h
E. coli
L
M
N
P=0.0016
P=0.0003
P=0.0035
P=0.0032
P=0.0014
P=0.0006
S. aureus
O
P
Q
Mock
L-ala
L-ala 0.01mM
L-ala 0.1mM
L-ala 1mM
P=0.0006
P=0.0006
P=0.5986
P=0.0004
P=0.0017
P=0.2331
P=0.0006
P=0.0001
P=0.0021
P=0.2475
WT
Prss1+/-
MPM, 24hpi

Supplementary Fig. 7 L-alanine interacts with Prss1 to induce NF-κB-mediated AMPs expression. (**A**) Structure of Biotin-L-alanine and Biotin-D-alanine. (**B**) Streptavidin pulldown assays of the binding of biotin-conjugated L-alanine to recombinant PRSS1. Unlabeled L-alanine or D-alanine were pretreated with PRSS1 for competition. (**C**) Domains of PRSS1. (**D**) Immunoblot and immunoprecipitation of lysates from HEK293T cells transfected with vector (-) or plasmids encoding full length FLAG-PRSS1(FL), Flag-PRSS1(N) or Flag-PRSS1(C). (**E**) Luciferase assay of HEK293T cells transfected with the NF-κB reporter gene with plasmids encoding PRSS1, TRAF6, TAK1, TAB1 or IKKα/β for 24 hours. (**F**) Immunoblot analysis of mice peritoneal macrophages pretreated with TAK1 inhibitor (5Z-7-Oxozeaenol, 5Z-7-OX) followed by H37Rv infection for 0, 3 and 6 hours (MOI=2). (**G-H**) Immunoblot analysis of wild type and *Prss1*$^{+/-}$ mice peritoneal macrophages infected with *Escherichia coli* (*E. coli*) (**G**) or *Staphylococcus aureus* (*S. aureus*) (**H**) for 0, 3 and 6 hours (MOI=2). (**I-K**) RT-PCR analysis of *Defb4* (**I**), *Defb3* (**J**) and *Camp* (**K**) in WT or *Prss1*$^{+/-}$ mice peritoneal macrophages infected with *E. coli* for 0, 3 and 6 hours (MOI=2). (**L-N**) RT-PCR analysis of *Defb4*(**L**), *Defb3* (**M**) and *Camp* (**N**) in WT or *Prss1*$^{+/-}$ mice peritoneal macrophages infected with *S. aureus* for 0, 3 and 6 hours (MOI=2). (**O-P**) RT-PCR analysis of *Defb3* (**O**) and *Camp* (**P**) in WT or *Prss1*$^{+/-}$ mice peritoneal macrophages treated with 1 mM L-alanine followed by infection with H37Rv for 24 hours (MOI=2). (**Q**) RT-PCR analysis of *Defb4* in WT or *Prss1*$^{+/-}$ mice peritoneal macrophages treated with L-alanine at different concentrations followed by infection with H37Rv for 24 hours (MOI=2). Data in **O**-**Q** represent fold change of AMPs compared with uninfected group. Data are representative of one experiment with at least three independent biological replicates; (**E, I-Q**) n = 3, each circle represents one technical repeat (mean ± s.e.m). Two-tailed unpaired Student's *t*-test (**E, I-Q**) was used for statistical analysis. *P* values are shown in **E** and **I-Q**. Source data are provided as a Source Data file.

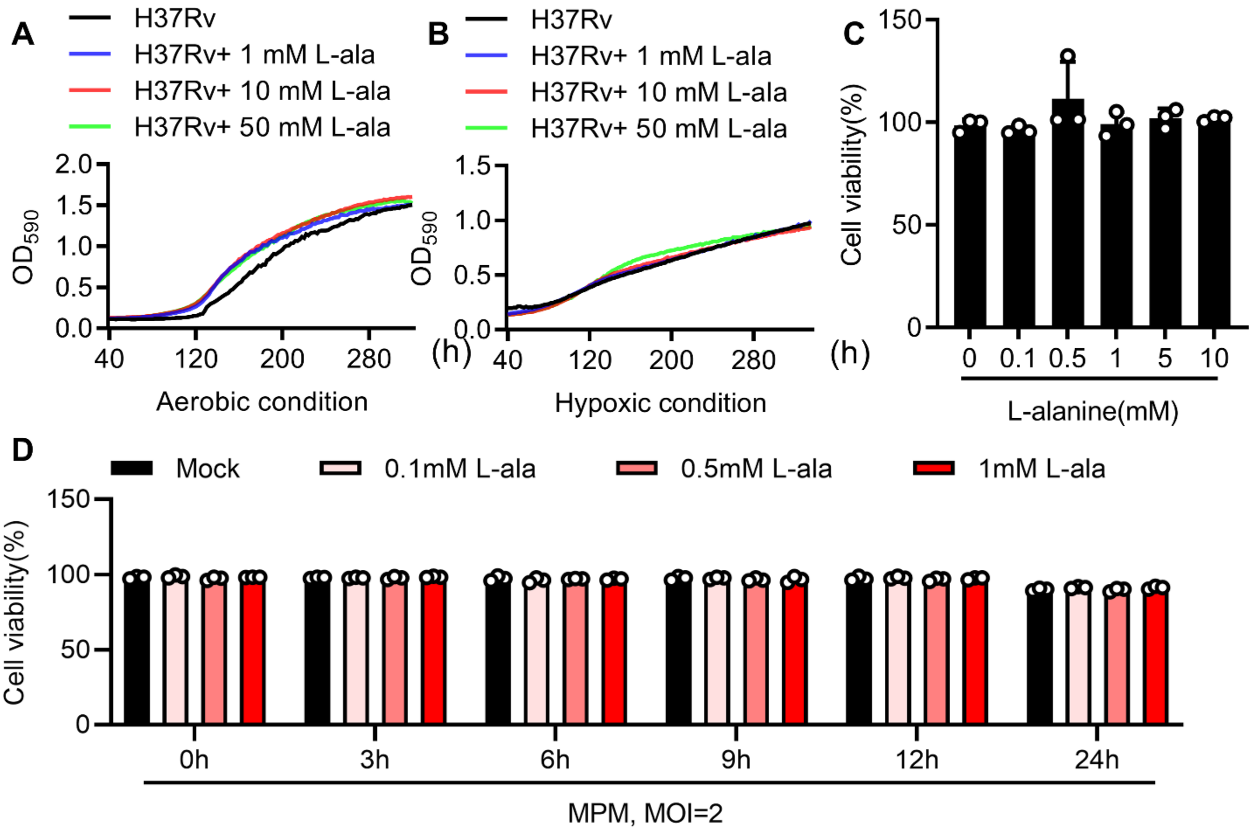

Supplementary Fig. 8 Targeting L-Alanine pathway for enhance anti-TB immunity. (**A-B**) Growth curve of H37Rv strains cultured with 1 mM, 10 mM, and 50 mM L-alanine in aerobic condition (**A**) and hypoxic condition (**B**). (**C**) MTT assay of cell viability from mice peritoneal macrophages treated with increasing concentration of L-alanine (0, 0.1, 0.5, 1, 5, 10 mM) for 24 hours. (**D**) MTT assay of cell viability from mice peritoneal macrophages treated with L-alanine at different concentrations followed by infection with H37Rv for 0, 3, 6, 9, 12 and 24 hours. Data are representative of one experiment with at least three independent biological replicates; (**C-D**) $n = 3$, each circle represents one technical repeat (mean ± s.e.m). Two-tailed unpaired Student's *t*-test (**C-D**) was used for statistical analysis. Source data are provided as a Source Data file.

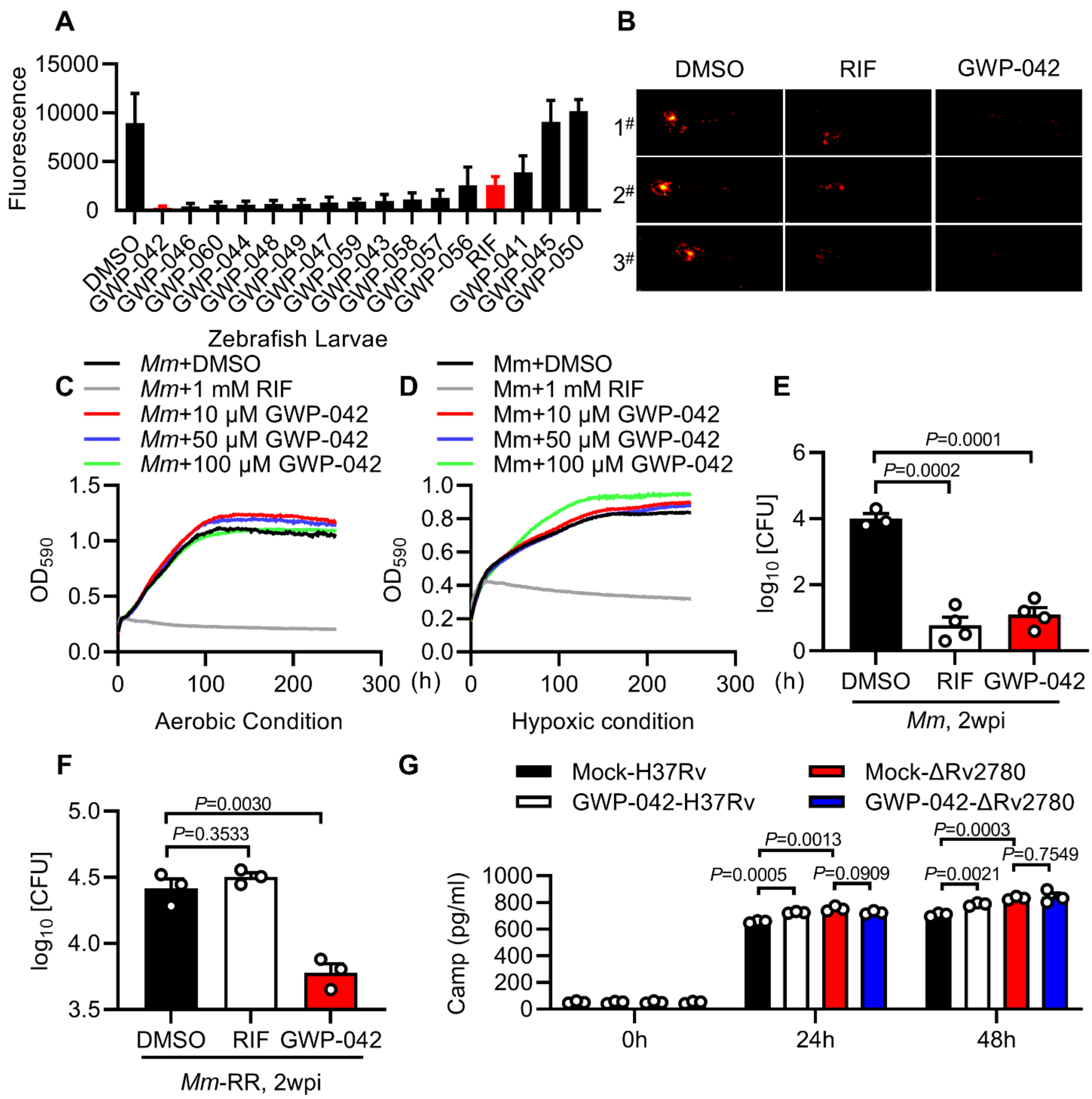

Supplementary Fig. 9 Targeting L-Alanine pathway for enhance anti-TB immunity. (**A**) Fluorescence quantification of Td-Tomato labeled *M. marinum* in zebrafish larvae treated with Rv2780 inhibitors or rifampicin (RIF). (**B**) Fluorescence images of zebrafish larvae infected with Td-Tomato labeled *M. marinum* for 7 days, followed by GWP-042 treatment for another 7 days. 1#, 2# and 3# represent representative pathological sections from 3 fish. (**C-D**) Growth curve of *Mycobacterium marinum* (*Mm*) strain cultured with 10 μM, 50 μM, and 100 μM GWP-042 or 1 mM RIF in aerobic condition (**C**) and hypoxic condition (**D**). RIF was used as positive control. (**E-F**) CFU assay in zebrafish infected with RIF sensitive (**E**) and RIF resistant (**F**) *M. marinum* (*Mm*-RR) for 7 days followed by treatment with GWP-042 for another 7 days. RIF was used as positive (**E**) and negative (**F**) control. (**G**) ELISA analysis of Camp in mice peritoneal macrophages treated with 50 μM GWP-042 followed by H37Rv or H37RvΔRv2780 infection for 0, 24 and 48 hours (MOI=2). Data are representative of one experiment with at least three independent biological replicates; (**A**) n = 2-16

zebrafish larva (mean ± s.e.m); (**E-F**) *n* = 3 zebrafish (mean ± s.e.m); (**G**) *n* = 3, each circle represents one technical repeat (mean ± s.e.m). Two-sided Mann–Whitney *U*-test (**E-F**) and two-tailed unpaired Student's *t*-test (**G**) was used for statistical analysis. *P* values are shown in **E-G**. Source data are provided as a Source Data file.

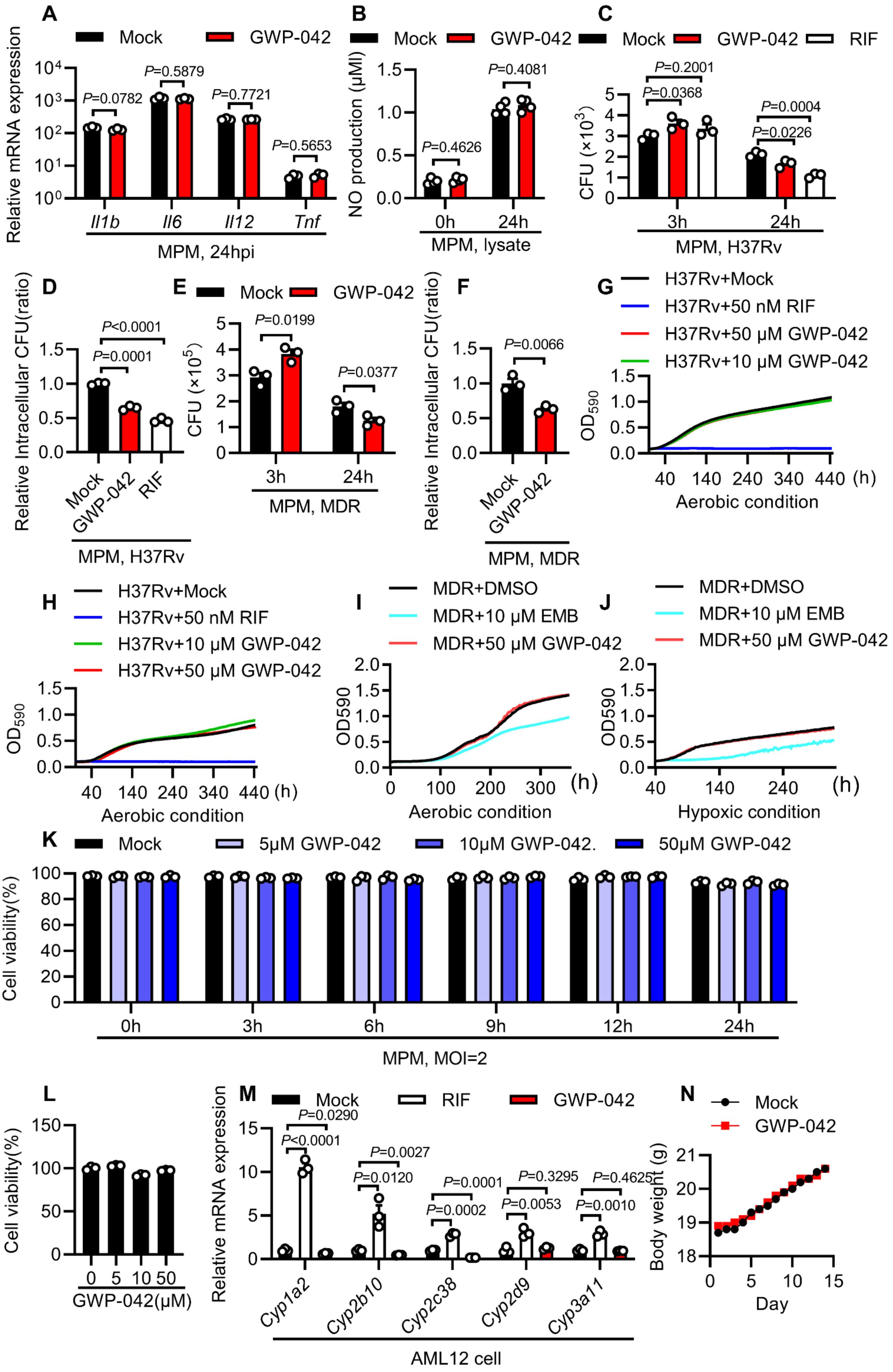
A
Mock
GWP-042
Relative mRNA expression
P=0.0782
P=0.5879
P=0.7721
P=0.5653
Il1b
Il6
Il12
Tnf
MPM, 24hpi
B
NO production (μMl)
P=0.4626
P=0.4081
0h
24h
MPM, lysate
C
RIF
CFU (×10^3)
P=0.2001
P=0.0368
P=0.0004
P=0.0226
3h
24h
MPM, H37Rv
D
Relative Intracellular CFU(ratio)
P<0.0001
P=0.0001
Mock
GWP-042
RIF
MPM, H37Rv
E
CFU (×10^5)
P=0.0199
P=0.0377
MPM, MDR
F
P=0.0066
MPM, MDR
G
H37Rv+Mock
H37Rv+50 nM RIF
H37Rv+50 μM GWP-042
H37Rv+10 μM GWP-042
OD590
Aerobic condition
H
I
MDR+DMSO
MDR+10 μM EMB
MDR+50 μM GWP-042
J
Hypoxic condition
K
5μM GWP-042
10μM GWP-042.
50μM GWP-042
Cell viability(%)
MPM, MOI=2
L
GWP-042(μM)
M
P=0.0290
P<0.0001
P=0.0027
P=0.0120
P=0.0001
P=0.0002
P=0.3295
P=0.0053
P=0.4625
P=0.0010
Cyp1a2
Cyp2b10
Cyp2c38
Cyp2d9
Cyp3a11
AML12 cell
N
Body weight (g)
Day

Supplementary Fig. 10 Targeting Rv2780 with GWP-042 enhances anti-TB immunity. (**A-B**) Quantitative analysis of *Il1b*, *Il6, Il12* and *Tnf* expression (**A**) and NO production (**B**) in mice peritoneal macrophages treated with 50μM GWP-042 followed by H37Rv infection infected with H37Rv for 24 hours (MOI=2). (**C-D**) CFU counts (**C**) and relative intracellular CFU ratio (**D**) in mice peritoneal macrophages treated with 50 μM GWP-042 or 50 nM rifampicin (RIF) followed by H37Rv infection for 3 and 24 hours (MOI=2). (**E-F**) CFU counts (**E**) and relative intracellular CFU ratio (**F**) in mice peritoneal macrophages treated with 50 μM GWP-042 followed by multidrug resistant *M. tuberculosis* (MDR) infection for 3 and 24 hours (MOI=2). (**G-H**) Growth curve of H37Rv cultured with 50 μM GWP-042 or 50 nM rifampicin (RIF) in aerobic condition (**G**) and hypoxic condition (**H**). (**I-J**) Growth curve of MDR cultured with 10 μM Ethambutol (EMB) or 50 μM GWP-042 in aerobic condition (**I**) and hypoxic condition (**J**). (**K**) MTT assay of cell viability from mice peritoneal macrophages treated with GWP-042 at different concentrations followed by infection with H37Rv for 0, 3, 6, 9, 12 and 24 hours. (**L**) MTT assay of cell viability from mice peritoneal macrophages treated with increasing concentration of GWP-042 (0, 5, 10, 50 μM) for 24 hours. (**M**) RT-PCR analysis of *Cyp* expression in AML12 cells treated with 10 μM RIF or 50 μM GWP-042 for 48 hours. (**N**) Body weight of 6-week-old BALB/c mice treated with GWP-042 in 15 days. Data are representative of one experiment with at least three independent biological replicates, mean in **G-J** and mean ± s.e.m in **A-F** and **K-N**. Two-tailed unpaired Student's t-test was used for statistical analysis in **A-F** and **M**. Data are representative of one experiment with at least three independent biological replicates; (**A-F** and **K-M**) $n = 3$, each circle represents one technical repeat (mean ± s.e.m); (**G-J** and **N**) (mean). Two-tailed unpaired Student's *t*-test (**A-F** and **M**) was used for statistical analysis. *P* values are shown in **A-F** and **M**. Source data are provided as a Source Data file.

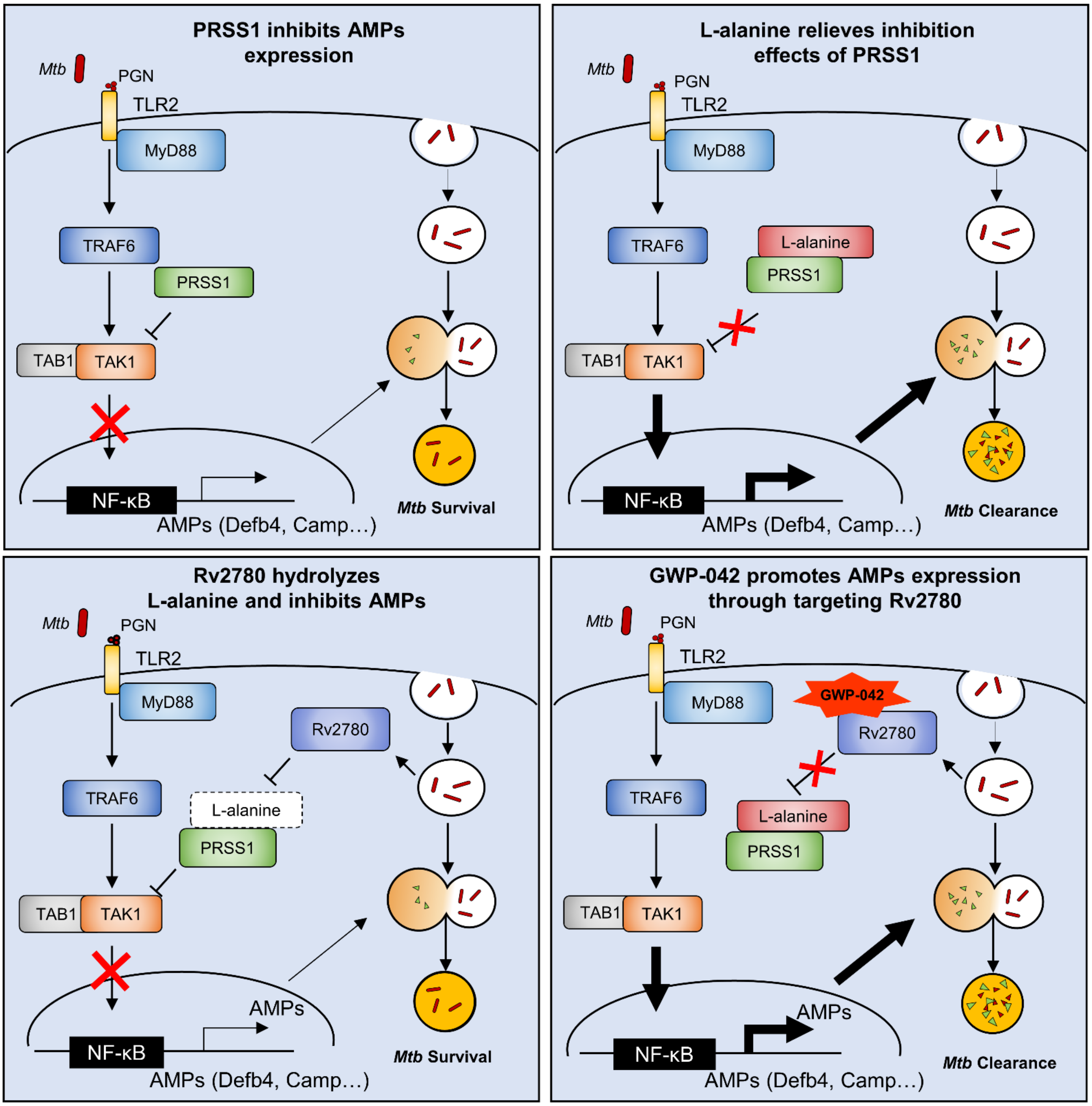

Supplementary Fig. 11 Diagram. 1). PRSS1 inhibits NF-κB-mediated expression of AMPs by disrupting the formation of the TAK1-TAB1 complex; 2). L-alanine directly interacts with PRSS1, which disabled the latter's inhibitory effect on TAK1/TAB1 complex formation, thereby triggering the NF-κB-mediated expression of AMPs; 3). *M. tuberculosis* secretes an alanine dehydrogenase that hydrolyzes L-alanine in host macrophages, thus suppressing the production of AMPs to facilitate the intracellular survival of mycobacteria; 4). Targeting mycobacterial Ald by GWP-042 resuscitates the production of AMPs to eliminate *M. tuberculosis*.

Supplementary Fig. 1A

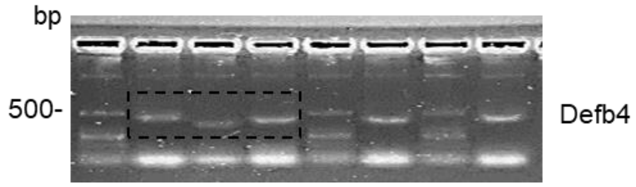

Supplementary Fig. 1F

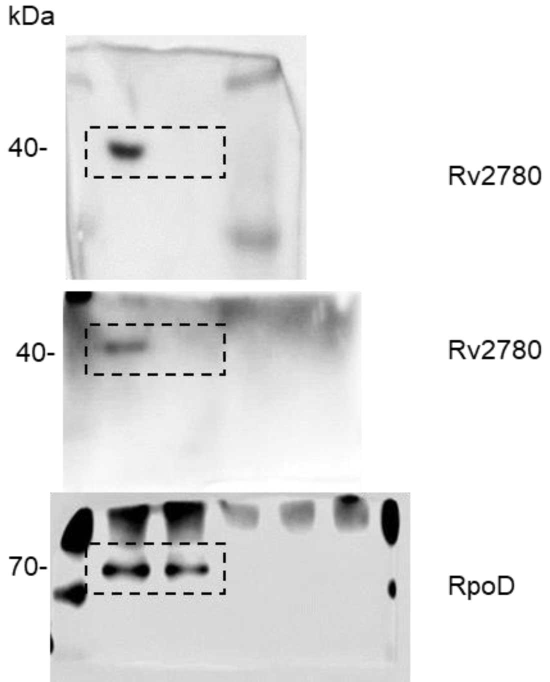

Supplementary Fig. 1G

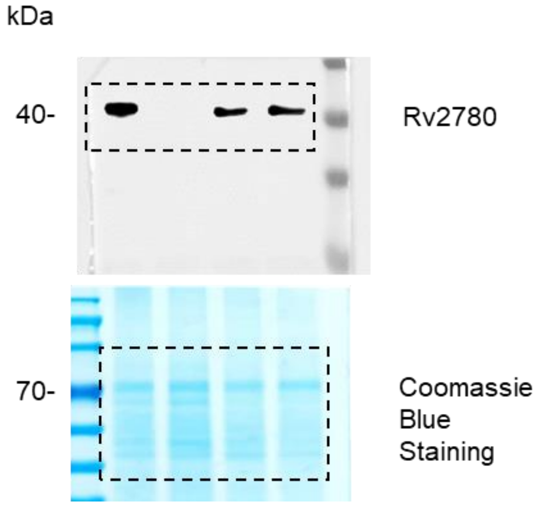

Supplementary Fig. 1H

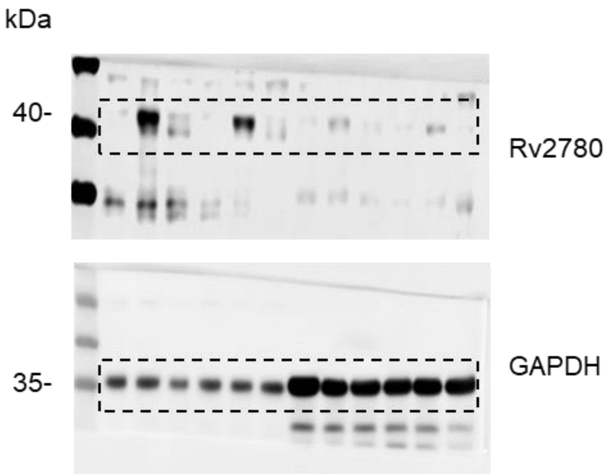

Supplementary Fig. 7B

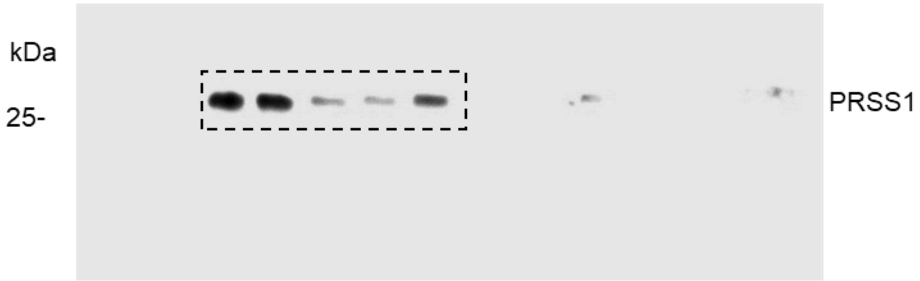

Supplementary Fig. 7D

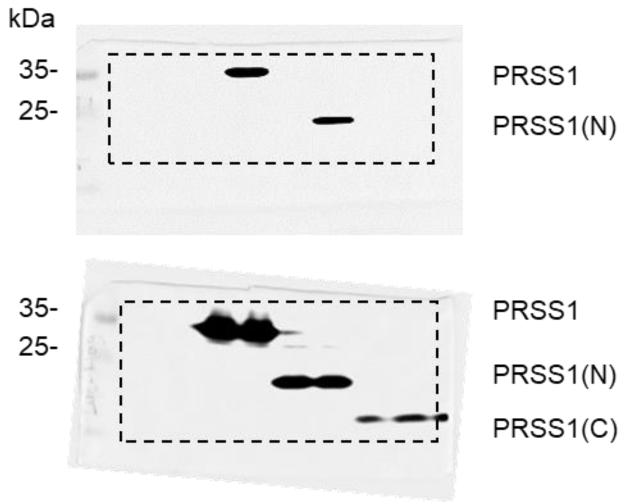

Supplementary Fig. 7F

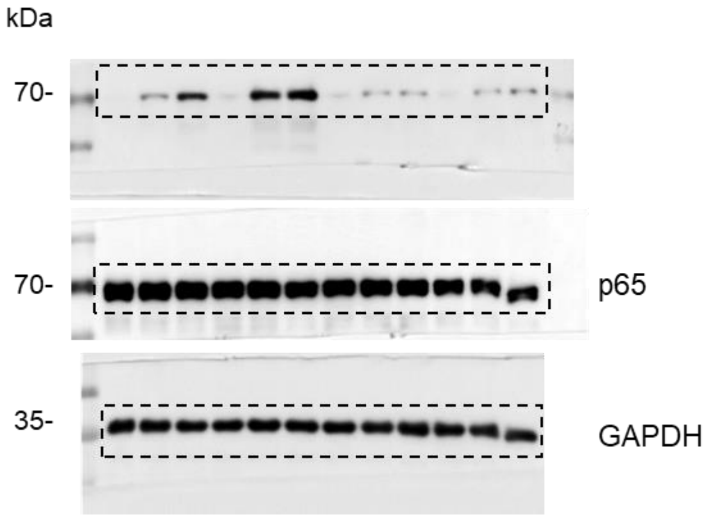

Supplementary Fig. 7G

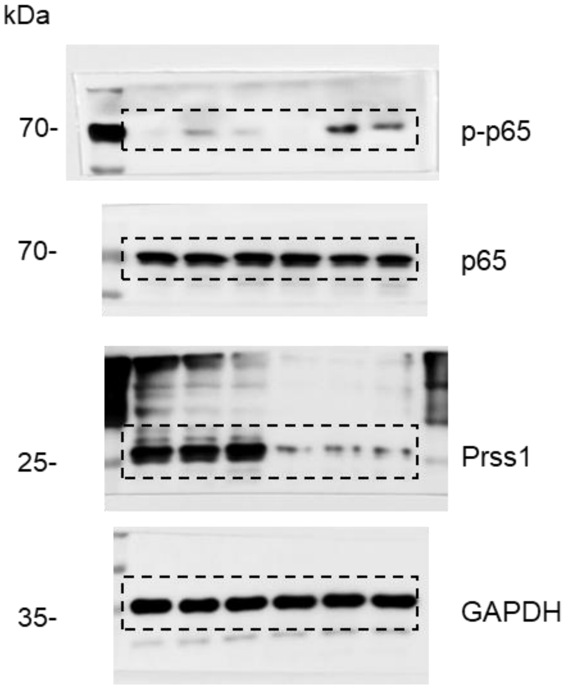

Supplementary Fig. 7H

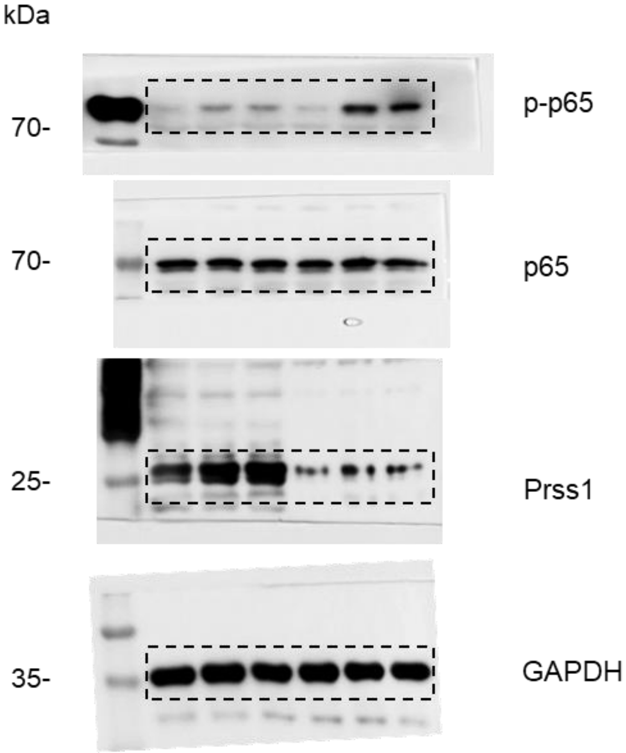

Supplement: Supplementary file 1 — Supplementary Information [file 41467_2024_48588_MOESM1_ESM.pdf]
