# Supplementary material for: Mycobacterium tuberculosis suppresses host antimicrobial peptides by dehydrogenating L-alanine
Source: Nat Commun. 2024 May 17;15:4216. doi: 10.1038/s41467-024-48588-4 (PMC11101664; doi:10.1038/s41467-024-48588-4)
Supplement: Supplementary file 3 — Description of Additional Supplementary Files [file 41467_2024_48588_MOESM3_ESM.pdf]

Description of Additional Supplementary Files

Supplementary Data 1 : Screening of 201 Mtb secreted proteins

Screening of *M. tuberculosis* proteins that inhibit the expression of antimicrobial peptide *DEFB4* expression by transfection of plasmids encoding 201 *M. tuberculosis* secreted protein in HEK293T cells and examination of relative *DEFB4* mRNA level using reverse transcription (RT)-PCR.

Supplementary Data 2 : Metabolite profiling by GC-MS (peak+area) in sera of Control and H37Rv-infected mice

Gas chromatography-mass spectroscopy analysis of metabolites in sera of C57BL/6J mice infected with *M. tuberculosis* H37Rv for 30 days. Control 1-4 represent four replicates of uninfected mice and H37Rv 1-4 represent four replicates of H37Rv-infected mice. Data represent peak and area of metabolites.

Supplementary Data 3 : Metabolite profiling by GC-MS (peak+area) in macrophages infected with different Mtb strains

Gas chromatography-mass spectroscopy analysis of metabolites in mice peritoneal macrophages infected with H37Rv, H37RvΔRv2780, H37RvΔRv2780+Rv2780 and H37RvΔRv2780+Rv2780^{DM} for 24 hours (MOI = 2). H37Rv 1-2, ΔRv2780 1-2, ΔRv2780+Rv2780 1-2 and H37RvΔRv2780+Rv2780^{DM} 1-2 represent two biological replicates from macrophages infected with indicated strains. Data represent peak and area of metabolites.

Supplementary Data 4 : Carbon metabolic flux in macrophages infected with different Mtb strains

Carbon metabolic flux of metabolites in mice peritoneal macrophages pretreated with U^{13}C glucose followed by infection with H37Rv and H37RvΔRv2780 for 24 hours (MOI = 2). H37Rv-24h 1-3 and ΔRv2780-24h 1-3 represent three biological replicates from macrophages infected with indicated strains for 24 hours. Data represent percentage of different isotopologues of metabolites.

Supplementary Data 5 : Biotin-L-alanine binding proteins idetified by MS

Mass spectrometry analysis of proteins in cell lysates of mice peritoneal macrophages incubated with biotin labeled L-alanine (Biotin-L-alanine) or biotin followed by streptavidin

pull down assay. Data shown represent proteins specifically identified in Biotin-L-alanine group compared with Biotin group.

Supplementary Data 6 : Top 50 compounds information of virtual screening

Top 50 compounds ranked according to the docking score of 309800 compounds to Rv2780 protein in virtual screening by molecular docking with 2D structures of compounds and 3D structure of MtAlaDH Rv2780 (PDB ID: 2VHX).

Supplementary Data 7 : Preliminary pharmacokinetic(PK) evaluation of GWP-042 in mice

Pharmacokinetic analysis of GWP-042 in C57BL/6J mice by intravenous injection (iv) or oral administration(op) of mice with GWP-042 followed by LC-MS/MS analysis of blood samples and evaluation of half-life ($T_{1/2}$), high maximal concentration (C_{max}) and clearance (CI).

Supplementary Data 8 : In vivo toxicity study of GWP-042

In vivo toxicity study of GWP-042 on C57BL/6J mice by administration with GWP-042 of 50mg/kg, 200mg/kg and 1000mg/kg respectively by oral gavage and observation of mortality and toxic signs for 14 days.

Supplementary Data 9 : Strains, plasmids and primers used in this study

Strains, plasmids and primers used in this study.
