# Supplementary material for: Mycobacterium tuberculosis suppresses host antimicrobial peptides by dehydrogenating L-alanine
Source: Nat Commun. 2024 May 17;15:4216. doi: 10.1038/s41467-024-48588-4 (PMC11101664; doi:10.1038/s41467-024-48588-4)
Supplement: Supplementary file 13 — Reporting Summary [file 41467_2024_48588_MOESM13_ESM.pdf]

nature portfolio

Corresponding author(s): Lin Wang[illegible] Baoxue Ge

Last updated by author(s): Apr 17, 2024

Reporting Summary

Nature Portfolio wishes to improve the reproducibility of the work that we publish. This form provides structure for consistency and transparency in reporting. For further information on Nature Portfolio policies, see our Editorial Policies and the Editorial Policy Checklist.

Statistics

For all statistical analyses, confirm that the following items are present in the figure legend, table legend, main text, or Methods section.

n/a	Confirmed	
☐	☒	The exact sample size (n) for each experimental group/condition, given as a discrete number and unit of measurement
☐	☒	A statement on whether measurements were taken from distinct samples or whether the same sample was measured repeatedly
☐	☒	The statistical test(s) used AND whether they are one- or two-sided<br>*Only common tests should be described solely by name; describe more complex techniques in the Methods section.*
☐	☒	A description of all covariates tested
☐	☒	A description of any assumptions or corrections, such as tests of normality and adjustment for multiple comparisons
☐	☒	A full description of the statistical parameters including central tendency (e.g. means) or other basic estimates (e.g. regression coefficient) AND variation (e.g. standard deviation) or associated estimates of uncertainty (e.g. confidence intervals)
☐	☒	For null hypothesis testing, the test statistic (e.g. F, t, r) with confidence intervals, effect sizes, degrees of freedom and P value noted<br>*Give P values as exact values whenever suitable.*
☒	☐	For Bayesian analysis, information on the choice of priors and Markov chain Monte Carlo settings
☒	☐	For hierarchical and complex designs, identification of the appropriate level for tests and full reporting of outcomes
☒	☐	Estimates of effect sizes (e.g. Cohen's d, Pearson's r), indicating how they were calculated

Our web collection on statistics for biologists contains articles on many of the points above.

Software and code

Policy information about availability of computer code

Data collection	The qRT-PCR data: ViiA 7 Software v1.2.4 on ViiA 7DX.<br>The western blot data: ImageQuant LAS 4000mini and Amersham Imager 600.<br>The enzymatic activity/ELISA/luciferase/Alanine/MTT assay/ROS/NO data: Tecan Infinite M200 Pro.<br>The confocal image: Leica SP8 confocal microscope.<br>The growth curve: BIOSCREEN FP-1100-C Automated Microbiology Growth Curve Analysis System.<br>The zebrafish larva imaging: Leica DMI4000 B microscope.
Data analysis	GraphPad Prism software 9.5.0 was used for all analyses.

For manuscripts utilizing custom algorithms or software that are central to the research but not yet described in published literature, software must be made available to editors and reviewers. We strongly encourage code deposition in a community repository (e.g. GitHub). See the Nature Portfolio guidelines for submitting code & software for further information.

Data

Policy information about availability of data

All manuscripts must include a data availability statement. This statement should provide the following information, where applicable:

- Accession codes, unique identifiers, or web links for publicly available datasets
- A description of any restrictions on data availability
- For clinical datasets or third party data, please ensure that the statement adheres to our policy

Data and materials availability:
Source Data in this study are available in Source Data file. Mtb secretory protein screening, metabolite profiling, carbon metabolic flux, mass spectrometry, preliminary pharmacokinetic evaluation and in vivo toxicity data in this study are available in Supplementary Data file. Further information and requests for resources or reagents should be directed to and will be fulfilled by Lin Wang (651377481@qq.com) or Baoxue Ge (gebaoxue@sibs.ac.cn).

Research involving human participants, their data, or biological material

Policy information about studies with human participants or human data. See also policy information about sex, gender (identity/presentation), and sexual orientation and race, ethnicity and racism.

Reporting on sex and gender	Our study does not·involve·sex- and gender-biased analyses.
Reporting on race, ethnicity, or other socially relevant groupings	Our study does not·involve·race, ethnicity, or other socially relevant groupings-biased analyses.
Population characteristics	All the TB patients and healthy volunteers shown in the study were 21-68 years old of both genders from Shanghai Pulmonary Hospital between 2020 and 2021.
Recruitment	Smear-positive TB patients:<br>Diagnostic criteria for TB were as follows: (1) sputum culture for Mtb; (2) presence of acid-fast bacilli in sputum smear; (3) clinical presentation and radiological signs (such as X-ray or computed tomography scan). The diagnosis was ultimately confirmed by culture of Mtb from sputum.
Ethics oversight	The ethics committee of Shanghai Pulmonary Hospital approved this consent procedure (permit number: K23-333Z).

Note that full information on the approval of the study protocol must also be provided in the manuscript.

Field-specific reporting

Please select the one below that is the best fit for your research. If you are not sure, read the appropriate sections before making your selection.

☒ Life sciences ☐ Behavioural & social sciences ☐ Ecological, evolutionary & environmental sciences

For a reference copy of the document with all sections, see nature.com/documents/nr-reporting-summary-flat.pdf

Life sciences study design

All studies must disclose on these points even when the disclosure is negative.

Sample size	No statistical methods were used to predetermine sample sizes. Sample size was based on empirical data from pilot experiments.
Data exclusions	No exclusion of data points or images were used.
Replication	All attempts at replication were successful as indicated in the figure legends.
Randomization	For animal experiments, mice were randomly chosen from 6-10 weeks old age-matched and sex-matched mice in each group. For cellular experiments, cells were randomly cultured in the plate, infection or drug treatment were randomly applied to the cells. For clinical sample analysis, data was compared by age-matched and sex-matched.
Blinding	In animal study, investigators were not blinded to infect mice with specific strains or treat mice with specific drugs to evaluate the lung bacterial burden and histopathology. These indexes are concrete indicator which are rarely influenced by subjective factors. In cellular experiments, investigators need to clarify the specific strains or treatments so they are not blinded to the experiments. RT-PCR/western blot/ELISA/Alanine detection and etc data were collected by software and may not be influenced by investigators.

Reporting for specific materials, systems and methods

We require information from authors about some types of materials, experimental systems and methods used in many studies. Here, indicate whether each material, system or method listed is relevant to your study. If you are not sure if a list item applies to your research, read the appropriate section before selecting a response.

Materials & experimental systems

n/a	Involved in the study
☐	☒ Antibodies
☐	☒ Eukaryotic cell lines
☒	☐ Palaeontology and archaeology
☐	☒ Animals and other organisms
☐	☒ Clinical data
☐	☒ Dual use research of concern
☒	☐ Plants

Methods

n/a	Involved in the study
☒	☐ ChIP-seq
☒	☐ Flow cytometry
☒	☐ MRI-based neuroimaging

Antibodies

Antibodies used

The polyclonal rabbit anti-Rv2780 antibody was produced and purified by ABclonal Biotech. The following antibodies were used in this study. Rabbit anti-HA antibody (H6908/polyclonal, Sigma-Aldrich, 1:2000 for immunoblot analysis); rabbit anti-GAPDH antibody (SAB2701826/polyclonal, Sigma-Aldrich, 1:2000 for immunoblot analysis) , rabbit anti-FLAG antibody (F7425, Sigma-Aldrich, 1:2000 for immunoblot analysis), Goat anti-Rabbit IgG (H+L) Secondary Antibody, Alexa Fluor 488 (A-11008, Invitrogen, 1:500 for immunofluorescence), anti-FLAG M2 Magnetic Beads (M8823, Sigma-Aldrich, for immunoprecipitation), rabbit Anti-PRSS1 antibody (ab200996/monoclonal, Abcam, 1:1000 for immunoblot analysis) , rabbit anti-NF-κB p65 (C22B4) antibody (4764/monoclonal, Cell Signaling Technology, 1:1000 for immunoblot analysis), rabbit anti-TAK1 (D94D7)antibody(5206/monoclonal, Cell Signaling Technology, 1:1000 for immunoblot analysis, 1:50 for immunoprecipitation), rabbit anti-TAB1 antibody (A5749/polyclonal, Abclonal Technology, 1:1000 for immunoblot analysis), rabbit anti-phospho-TAK1 (Thr187) antibody (4536/polyclonal, Cell Signaling Technology, 1:1000 for immunoblot analysis), rabbit anti-phospho-NF-κB p65 (Ser536) antibody(3033/monoclonal, Cell Signaling Technology, 1:1000 for immunoblot analysis), purified anti-E. coli RNA Sigma 70 antibody(663202, BioLegend, 1:1000 for immunoblot analysis of RpoD). They are described in Methods section (Plasmids, antibodies and reagents).

Validation

Validation information:
Rabbit anti-HA antibody (H6908/polyclonal, Sigma-Aldrich, 1:2000 for immunoblot analysis): https://www.sigmaaldrich.cn/CN/en/product/sigma/h6908
Rabbit anti-GAPDH antibody (SAB2701826/polyclonal, Sigma-Aldrich): https://www.sigmaaldrich.cn/CN/en/product/sigma/sab2701826
Rabbit anti-FLAG antibody (F7425, Sigma-Aldrich, 1:2000 for immunoblot analysis): https://www.sigmaaldrich.cn/CN/en/product/sigma/f7425
Anti-FLAG M2 Magnetic Beads (M8823, Sigma-Aldrich): https://www.sigmaaldrich.cn/CN/en/product/sigma/m8823
Rabbit Anti-PRSS1 antibody (ab200996/monoclonal, Abcam): https://www.abcam.com/products/primary-antibodies/trypsin-antibody-epr19497-ab200996.html
Rabbit anti-NF-κB p65 (C22B4) antibody (4764/monoclonal, Cell Signaling Technology): https://www.cellsignal.cn/products/experimental-controls/bcl-xl-blocking-peptide/4764
Rabbit anti-TAK1 (D94D7)antibody(5206/monoclonal, Cell Signaling Technology, 1:1000 for immunoblot analysis): https://www.cellsignal.cn/products/primary-antibodies/tak1-d94d7-rabbit-mab/5206
Rabbit anti-TAB1 antibody (A5749/polyclonal, Abclonal Technology, 1:1000 for immunoblot analysis): https://abclonal.com.cn/catalog/A5749
Rabbit anti-phospho-TAK1 (Thr187) antibody (4536/polyclonal, Cell Signaling Technology): https://www.cellsignal.cn/products/primary-antibodies/phospho-tak1-thr187-antibody/4536
Rabbit anti-phospho-NF-κB p65 (Ser536) antibody (3033/monoclonal, Cell Signaling Technology): https://www.cellsignal.cn/products/primary-antibodies/phospho-nf-kb-p65-ser536-93h1-rabbit-mab/3033
Purified anti-E. coli RNA Sigma 70 antibody (663208, BioLegend): https://www.biolegend.com/en-gb/products/purified-anti-e-coli-rna-sigma-70-antibody-18128

Eukaryotic cell lines

Policy information about cell lines and Sex and Gender in Research

Cell line source(s)

HEK293T cells (ATCC CRL-3216), A549(ATCC CRM-CCL-185) and AML-12 cells (ATCC CRL-2254) were obtained from the American type culture collection (ATCC) as described in Materials and Methods section (Cell Culture part).

Authentication

Cell lines purchased from commercial vendors, ATCC have been authenticated by the commercial vendor using short tandem repeat (STR) analysis.

Mycoplasma contamination

All the cells were routinely tested for contamination by mycoplasma as described in Methods section (Cell Culture part). All cell lines used tested negative.

Commonly misidentified lines
(See ICLAC register)

No commonly misidentified cell lines were used in this study.

Animals and other research organisms

Policy information about studies involving animals; ARRIVE guidelines recommended for reporting animal research, and Sex and Gender in Research

Laboratory animals	Prss1+/- mice on C57BL/6J genetic background were purchased from the Cyagen Biosciences. Macrophage conditional knockout mice Female 6-8 weeks old SPF C57BL/6J and SCID mice were purchased from Slaccas for peritoneal macrophages and BMDMs separation. Prss1floxp/floxp mice and Lyz2cre mice on C57BL/6J genetic background were purchased from Shanghai Model Organisms Center. Macrophage conditional Prss1 knockout mice were generated by breeding Prss1floxp/floxp mice and Lyz2cre mice.<br>All the mice infection experiments were performed with age- and sex- matched groups of 8–12-weeks old mice. All mice were maintained at the animal faclity of Tongji University Animal Experimental center under 12-hour light dark cycle at 20-24°C and 45-65% humidity. Animals were housed with a maximum of 5 mice per cage Female mice were only used for breeding.<br>Zebrafish AB wild-type strain was obtained from EzeRinka Biotech. The fishes were reared in recirculating fish systems obtained from Qingdao Elvin Marine Technology Co., Ltd. (Qingdao, China), and transferred to a flowthrough fish system for the infection experiment. Up to 10 fish were kept in a 3L tank and tanks were maintained under standard conditions for housing zebrafishes (water temperature ~28°C, pH ~7.4, and conductivity ~1500µS). Zebrafish larvae experiments were conducted with zebrafish larvae at 48 hours post fertilization. Adult zebrafish experments were conducted with 2 month-old adult zebrafishes.
Wild animals	The study did not involve wild animals.
Reporting on sex	Both gender of mice were used in this study.
Field-collected samples	The study did not involve samples collected from the field.
Ethics oversight	All animal experiments were reviewed and approved by the Animal Experiment Administration Committee of Shanghai Pulmonary Hospital and were conducted in accordance with the National Institutes of Health (NIH) Guidelines for the Care and Use of Laboratory Animals.

Note that full information on the approval of the study protocol must also be provided in the manuscript.

Clinical data

Policy information about clinical studies

All manuscripts should comply with the ICMJE guidelines for publication of clinical research and a completed CONSORT checklist must be included with all submissions.

Clinical trial registration	N/A
Study protocol	N/A
Data collection	N/A
Outcomes	N/A

Dual use research of concern

Policy information about dual use research of concern

Hazards

Could the accidental, deliberate or reckless misuse of agents or technologies generated in the work, or the application of information presented in the manuscript, pose a threat to:

No	Yes	
☒	☐	Public health
☒	☐	National security
☒	☐	Crops and/or livestock
☒	☐	Ecosystems
☒	☐	Any other significant area

Experiments of concern

Does the work involve any of these experiments of concern:

No	Yes	
☒	☐	Demonstrate how to render a vaccine ineffective
☒	☐	Confer resistance to therapeutically useful antibiotics or antiviral agents
☒	☐	Enhance the virulence of a pathogen or render a nonpathogen virulent
☒	☐	Increase transmissibility of a pathogen
☒	☐	Alter the host range of a pathogen
☒	☐	Enable evasion of diagnostic/detection modalities
☒	☐	Enable the weaponization of a biological agent or toxin
☒	☐	Any other potentially harmful combination of experiments and agents

Plants

Seed stocks	N/A
Novel plant genotypes	N/A
Authentication	N/A
